# Acute Skeletal Muscle Wasting is Associated with Prolonged Hospital Stay in Critical Illness with Brain Injury

**DOI:** 10.1007/s12028-024-02017-y

**Published:** 2024-06-25

**Authors:** Melda Kangalgil, Hülya Ulusoy, Sekine Ayaz

**Affiliations:** 1 Department of Nutrition and Dietetics, Faculty of Health Sciences, Sivas Cumhuriyet University, Sivas, Turkey; 2 Department of Anesthesiology and Reanimation, Faculty of Medicine, Karadeniz Technical University, Trabzon, Turkey; 3 Department of Anesthesiology and Reanimation, Pasinler Ibrahim Hakkı State Hospital, Erzurum, Turkey

**Keywords:** Brain injury, Muscle wasting, Intensive care

## Abstract

**Background:**

Acute muscle wasting is common in critically ill patients, and this can lead to unfavorable clinical outcomes. The aim of this study was to identify factors associated with muscle wasting and to investigate the association between skeletal muscle wasting and prolonged hospital stay in critically ill patients with acute brain injury.

**Methods:**

This single-center prospective observational study was conducted in critically ill patients with acute brain injury who stayed in the intensive care unit for at least 1 week. The rectus femoris cross-sectional area was measured via ultrasound at baseline and a week after the first assessment. Univariate and multivariate logistic regression analyses were performed to identify factors that predicted prolonged hospital stay.

**Results:**

A total of 86 patients were included in the study. Their mean age was 49.4 ± 16.9 years, 57% were male, and 46.5% had an admission diagnosis of subarachnoid hemorrhage. The percentage change in the rectus femoris cross-sectional area was 15.8% (95% confidence interval [CI] − 19.8% to − 12.0%; *p* < 0.001), and 57% of all patients had acute muscle wasting. According to the univariate analysis, there was a significant association between prolonged hospital stay and acute muscle wasting (odds ratio [OR] 3.677; 95% CI 1.487–9.043; *p* = 0.005), mechanical ventilation status (OR 3.600; 95% CI 1.455–8.904; *p* = 0.006), and Glasgow Coma Scale score (OR 0.888; 95% CI 0.808–0.976; *p* = 0.014) at intensive care unit admission. The multivariate analysis demonstrated that acute muscle wasting (OR 3.449; 95% CI 1.344–8.853; *p* = 0.010) was an independent risk factor for prolonged hospital stay.

**Conclusions:**

There was considerable muscle wasting in critically ill patients with brain injuries over a 1-week period. Acute muscle wasting was associated with prolonged hospital stay in critically ill patients with acute brain injury.

## Introduction

An acute brain injury is defined as an acute cerebral disorder consequent to trauma or to a cerebrovascular event with high morbidity and mortality [1–3]. Critically ill patients with acute brain injury suffer a hypercatabolic state caused by stress-related and proinflammatory cytokines and hormones that can contribute to protein catabolism in skeletal muscles and muscle wasting [4, 5]. Muscle wasting begins early during critical illness, and patients can lose nearly 2% of skeletal muscle per day during the first week [6]. Although the pathophysiology of skeletal muscle wasting is related to several physiological processes, including insulin resistance, inflammation, oxidative stress, and mitochondrial damage, it is not fully understood [7]. Critically ill patients with brain injury have a high severity of illness requiring prolonged immobilization, mechanical ventilation, and higher doses of sedative medications, all of which are independent risk factors for muscle wasting [7, 8]. Moreover, brain injury causes an increase in catabolism, and this may lead to inadequate nutrition [9]. Therefore, patients with brain injury may be more susceptible to the risk of muscle wasting, but there are limited data on this condition [10–12].

To diagnose muscle wasting and weakness, ideally, clinical measurement of muscle strength should be performed. However, for this to be implemented, patients must be awake and cooperative and must understand the assessor’s instructions. Because patients are often unconscious or uncooperative, such an evaluation is often not possible, particularly with the diagnosis of muscle wasting in critically ill patients with brain injury [13]. However, various imaging methods are available for the evaluation of skeletal muscle, each with advantages and disadvantages [14, 15]. Of these methods, muscle ultrasound is being discussed with growing interest as a noninvasive tool to assess and track changes in the muscles of critically ill patients. The quadriceps is the most commonly measured region to assess muscle loss via ultrasound in these patients [16–18]. Previous studies have reported quadriceps muscle ultrasound as a potential tool for prediction of clinical outcomes of critical illness [19, 20]. However, whether the diagnosis of muscle wasting based on skeletal muscle ultrasound measurement is associated with clinical outcomes requires further investigation.

The aim of in the present study was to assess the association between acute muscle wasting and prolonged hospital stay in critically ill patients with acute brain injury. The secondary aim was to investigate clinical and nutritional parameters associated with acute muscle wasting and prolonged hospital stay.

## Methods

### Study Design and Patients

This prospective observational study was conducted in the adult intensive care unit (ICU) of a tertiary care university hospital between January 2020 and June 2022. The Strengthening the Reporting of Observational Studies in Epidemiology (STROBE) statement was used to organize and report the results [21]. The study protocol was approved by the Ethics Committee of Karadeniz Technical University. The procedures were in agreement with the Declaration of Helsinki, and written informed consent was obtained from all the participants or their relatives.

Adult critically ill patients with an acute brain injury defined as an acute cerebral disorder consequent to trauma or to a cerebrovascular event (subarachnoid hemorrhage, intracerebral hemorrhage, traumatic brain injury, acute ischemic stroke) and expected to stay in the ICU for at least 7 days were included. The exclusion criteria were based on identifying acute muscle wasting in the ICU and were aimed to exclude potential patient who had experienced or may experience muscle loss due to different mechanisms. The exclusion criteria were as follows: age < 18 years and > 90 years; other critically ill neurological populations (e.g., brain tumor, status epilepticus, anoxic-ischemic brain injury); expected to die or decision taken for withdrawal of life-sustaining therapy; pregnancy; body mass index > 35 kg/m^2^; the presence of diseases that may lead to chronic mobility impairment or muscle wasting at baseline, such as a prior disabling cerebrovascular event or primary systemic neuromuscular disease; amputated lower limbs; burn injury; malignancy; kidney failure with dialysis dependence; severe chronic liver disease or acute liver failure; history of ICU admission within the previous 6 months; a transfer record from an ICU and palliative care unit; and enrollment in an intervention study affecting the usual care process.

### Data Collection

The demographic and clinical characteristics, including the patients’ age, sex, type of brain injury, comorbidity status and Charlson Comorbidity Index score [22], Acute Physiology and Chronic Health Evaluation II score [23], Sequential Organ Failure Assessment score [24], and Glasgow Coma Scale (GCS) [25] score, were recorded. The modified Nutrition Risk in the Critically Ill score (in which a score of > 4 is high nutrition risk) was calculated to assess nutritional risk [26]. Daily cumulative doses of analgesic, sedative, and neuromuscular blocking agents were collected using drug administration records, and they were converted to equivalents [27]. Clinical outcomes were assessed based on the duration of mechanical ventilation, the length of the ICU and hospital stay, and ICU mortality. The length of hospital stay was defined as the period from admission to the ICU until discharge to home or the palliative care unit or until death.

### Dietary Assessment

Daily caloric and protein intake was recorded from all sources, including total parenteral nutrition, enteral nutrition, and oral nutrition, for a week. Intravenous energy was received from parenteral nutrition as well as from nonnutritional energy sources (dextrose solutions or propofol). Daily oral intake was evaluated with dietary recalls. The software Nutrition Knowledge System [28] was used to calculate daily energy and protein intakes from the total food and fluids consumed. Energy requirements were calculated using the Harris–Benedict equation [29]. Protein requirements were calculated according to the European Society for Clinical Nutrition and Metabolism guidelines for ICU patients [30]. Daily energy and protein adequacy (percentage) was calculated as follows: (daily amount delivered)/(daily estimated requirements) × 100. Adequacy over the study period was calculated as the total adequacy divided by the number of study days. Mean energy and protein delivered per kilogram of actual body weight averaged per study day were recorded.

### Muscle and Anthropometric Measurements

All muscle and anthropometric measurements were conducted on the right side of the body when possible, as per International Standards for Anthropometric Assessment, at baseline (within 48 h of ICU admission) and on study day 7. When this was not possible, the left side of the body was used for measurements. Body weight was measured using the weighing scales of the ICU beds. Height and body weight were used to calculate the body mass index. Mid-arm circumference (cm) was measured with the aid of an inelastic tape by marking the midpoint between the acromion and olecranon protrusion of the patient. Calf circumference (cm) was measured at the largest circumference of the leg. The mean of three consecutive measurements was recorded. The rectus femoris cross-sectional area (RFCSA) (cm^2^) was measured by B-mode ultrasonography (Esaote MyLab) using a variable-band linear array 13–4 MHz probe with 50 mm (Esaote LA523) by a single intensive care physician. Validation of muscle ultrasound measurements by the intensive care physician was compared with that by a radiology physician. The RFCSAs of 20 critically ill adult patients were separately measured by both physicians blinded to patient information. The interobserver reliability analysis was high (*r* = 0.942; *p* < 0.001). The measurement was performed in accordance with the protocols previously described in the ICU setting [31]. Ultrasound measurements were obtained with the transducer placed perpendicular to the long axis of the thigh at the upper two thirds of the length between the anterior superior iliac spine and the upper pole of the patella. Measurements were performed by applying minimal compression to the ultrasound probe. The inner echogenic line of the rectus femoris was traced manually on a frozen image, and the RFCSA was calculated. A decrease in the RFCSA of ≥ 10% was considered to define muscle wasting in accordance with previous studies in critically ill patients [32, 33].

### Statistical Analyses

There were no data on muscle wasting and clinical outcomes in critically ill patients with acute brain injury. Therefore, our sample size calculation was based on acute muscle loss. Previous studies of changes in the RFCSA over 1 week in critically ill patients have reported a wide range of effect sizes, from 0.2 to 0.8 [34–36]. We calculated the sample size as 84 patients using the software G*Power [37], with an α value of 0.05, a power of 0.95, and an effect size of 0.4. All statistical analyses were performed using SPSS (version 21; IBM). Categorical data were presented as count (percentage), and continuous data were reported as mean ± standard deviation or median (interquartile range [IQR]) as appropriate. Normality was assessed using the Kolmogorov–Smirnov test and confirmed by visual inspection of the histogram. For the comparison between measurements performed in the first and second assessments, a paired *t*-test was used. Normally distributed data were analyzed using Student’s *t*-test, and nonnormally distributed data were analyzed using the Mann–Whitney *U*-test. Logistic regression analysis was used to identify independent risk factors for prolonged stay. Variables with a *p* value lower than 0.2 in the univariate analysis were included in the multivariable regression analysis. All statistical tests were considered statistically significant at a two-tailed *p* value < 0.05.

## Results

### Clinical and Demographic Characteristics of the Patients

**Fig. 1:**
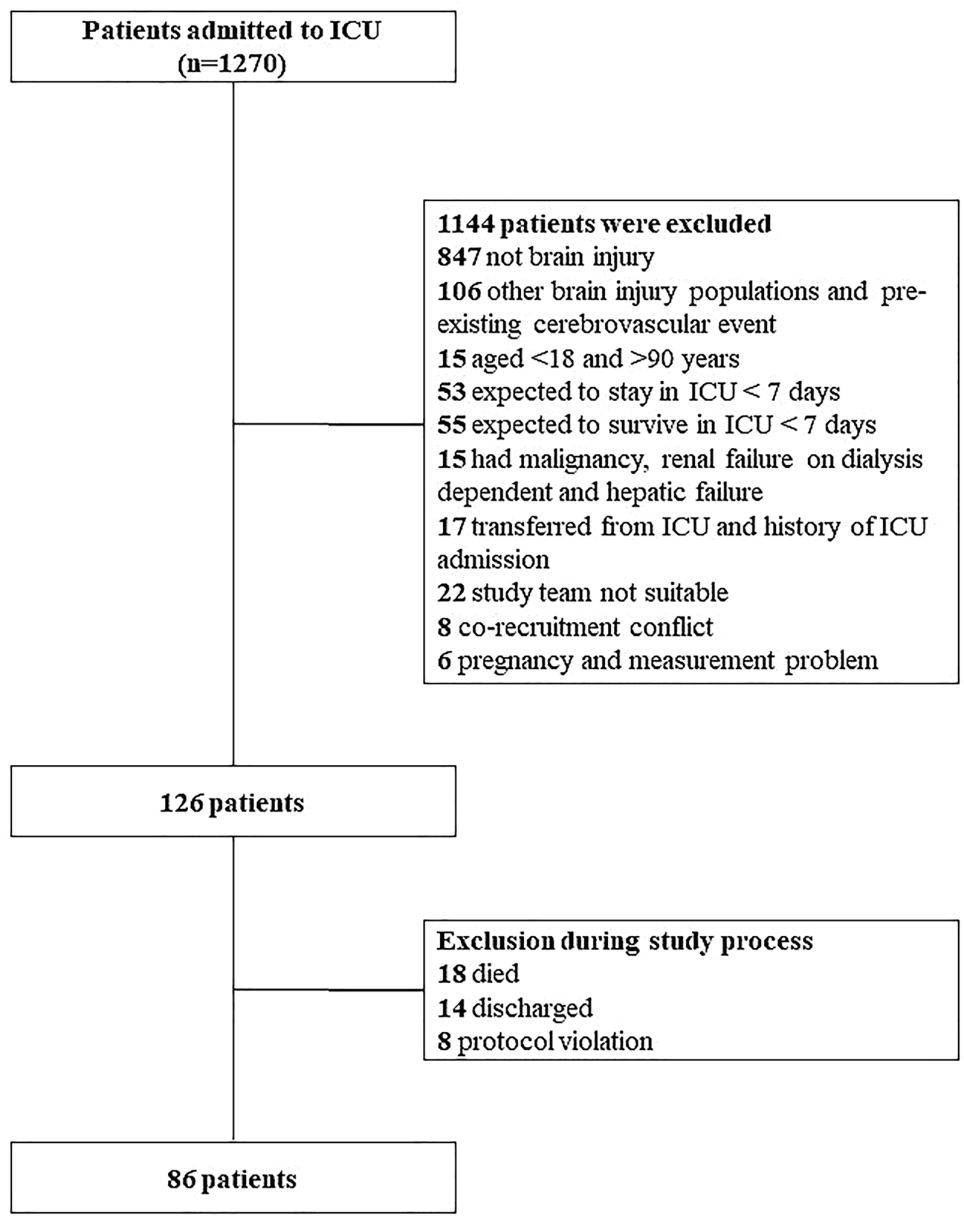
Flowchart of the inclusion of patients. *ICU* intensive care unit

A total of 1,270 patients were screened, and 126 patients were included in the study; 40 patients were excluded because of death, discharge, or protocol violation, leaving a total of 86 patients (Fig. 1). The patient demographics and clinical characteristics are presented in Table 1. The mean age was 49.4 ± 16.9 years, and 57% were male. The GCS score on admission to the ICU was 10 (5–15), and the most common admission diagnosis was subarachnoid hemorrhage (46.5%). The median (IQR) durations of mechanical ventilation and ICU and hospital stay were 4 (0–20), 16 (12–23), and 23 (15–31.7) days, respectively.

**Table 1:** Demographic and clinical characteristics of critically ill patients with acute brain injury

Characteristics	All patients (*N* = 86)	Decrease of RFCSA		*p* value	Hospital LOS		*p* value
		≥ 10% (*n* = 49)	< 10% (*n* = 37)		≥ 21 days (*n* = 50)	< 21 days (*n* = 36)	
Age, years, mean ± SD	49.4 ± 16.9	49.8 ± 18.8	48.9 ± 14.4	0.804	49.5 ± 16.9	49.4 ± 17.2	0.986
Sex, *n* (%)							
Female	37 (43)	21 (42.9)	16 (43.2)	0.572	19 (38)	18 (50)	0.280
Male	49 (57)	28 (57.1)	21 (56.8)		31 (62)	18 (50)	
Comorbidity, *n* (%)							
Yes	44 (51.2)	28 (57.1)	16 (43.2)	0.276	27 (54)	17 (47.2)	0.662
CCI, median (IQR)	1 (0–2)	1 (0–2.5)	1 (0–2)	0.649	1 (0–2)	0.5 (0–2)	0.553
Type of brain injury, *n* (%)							
TBI	35 (40.7)	20 (40.8)	15 (40.5)	0.168	21 (42)	14 (38.9)	0.840
SAH	40 (46.5)	20 (40.8)	20 (54.1)		22 (44)	18 (50)	
ICH	11 (12.8)	9 (18.4)	2 (5.4)		7 (14)	4 (11.1)	
APACHE-II, median (IQR)	10 (3–16.2)	10 (3–16.5)	9 (4–17)	0.865	10	8.5 (2.2–15.5)	0.393
SOFA, median (IQR)	4 (2–5)	4 (2–5)	3 (1–5)	0.788	4 (2–5)	2 (1–4.5)	0.131
GCS, median (IQR)	10 (5–15)	8 (4.5–14.5)	13 (5–15)	0.277	7 (4–14)	14 (6–15)	0.006
mNUTRIC, *n* (%)							
Low risk	71 (82.6)	41 (83.7)	30 (81.1)	0.781	43 (86)	28 (77.8)	0.392
Muscle parameters, mean ± SD							
Baseline RFCSA	4.1 ± 1.4	4.3 ± 1.5	3.8 ± 1.2	0.104	4.2 ± 1.3	4.0 ± 1.5	0.513
Daily dose of sedative or analgesic agent, median (IQR)							
Benzodiazepines (mg)	0 (0–3)	0 (0–3.9)	0 (0–1.0)	0.186	0.1 (0–4.2)	0 (0–0.2)	0.031
Propofol (mg)	0 (0–0)	0 (0–0)	0 (0–0)	0.263	0 (0–0)	0 (0–0)	0.324
Dexmedetomidine (μg)	10 (0–124.5)	39 (0–202.5)	0 (0–61.5)	0.017	33.5 (0–159)	0 (0–67)	0.162
Opiates (mg)	3.1 (0–11.1)	4.6 (0–11.1)	1 (0–12.5)	0.193	5.3 (0–14)	1.2 (0–5.8)	0.048
Daily dose of NMBA, median (IQR)							
Rocuronium (mg)	0 (0–0)	0 (0–0)	0 (0–0)	0.307	0 (0–0)	0 (0–0)	0.940
Baseline parameters							
Albumin, mean ± SD or median (IQR)	37.3 ± 5.7	37.3 ± 5.7	37.1 ± 5.6	0.893	37.8 (35–41.1)	36.2 (33.1–43.1)	0.358
Hemoglobin, mean ± SD	12.4 ± 1.9	12.3 ± 2.0	12.6 ± 1.6	0.446	12.5 ± 2.0	12.4 ± 1.7	0.702
Sodium, mean ± SD	138.4 ± 3.8	138.8 ± 3.6	137.6 ± 4.0	0.144	139 ± 3.6	137 ± 3.6	0.008
CRP, median (IQR)	14.6 (7.3–48.6)	14 (6.4–45.2)	17.7 (8.2–56.4)	0.350	17.3 (8.5–58.2)	12.4 (6.9–19.8)	0.102
Procalcitonin, median (IQR)	0.1 (0–.5)	0.1 (0–0.7)	0.1 (0.1–0.5)	0.882	0.2 (0.1–1.4)	0.1 (0–0.4)	0.414
Creatinine, median (IQR)	0.7 (0.6–0.9)	0.7 (0.6–1.0)	0.7 (0.5–0.9)	0.504	0.8 (0.6–1.0)	0.7 (0.5–0.8)	0.606
WBC, mean ± SD	13.3 ± 4.3	13.5 ± 4.4	12.8 ± 4.1	0.469	13.1 ± 4.4	13.6 ± 4.2	0.500
Platelet count, mean ± SD	211.6 ± 70.8	219.4 ± 69.0	198.8 ± 69.7	0.177	218.8 ± 65.6	202.4 ± 77	0.373

*APACHE-II* Acute Physiology and Chronic Health Evaluation II, *CCI* Charlson Comorbidity Index, *CRP* C-reactive protein, *GCS* Glasgow Coma Scale, *ICH* intracranial hemorrhage, *IQR* interquartile range, *LOS* length of stay, *mNUTRIC* modified Nutrition Risk in the Critically Ill, *NMBA* neuromuscular blocking agent, *RFCSA* rectus femoris cross-sectional area, *SAH* subarachnoid hemorrhage, *SOFA* Sequential Organ Failure Assessment, *TBI* traumatic brain injury, *WBC* white blood cell count

### Association Between Muscle Wasting, Prolonged Hospital Stay, and Clinical Parameters

Time to baseline ultrasound measurement was a median (IQR) of 1 (1–1) day following ICU admission. The RFCSA decreased significantly from study days 1 to 7 (15.8%; 95% confidence interval [CI] − 19.8% to − 12.0%; *p* < 0.001). A decrease in the RFCSA of ≥ 10% was seen in 57% of all patients. Patients with a lower median GCS score at admission to the ICU had a longer hospital stay (*p* = 0.006). There was no difference in age, sex, comorbidity, type of brain injury, and Acute Physiology and Chronic Health Evaluation II, Sequential Organ Failure Assessment, and modified Nutrition Risk in the Critically Ill scores between the groups. The sedative or analgesia agent taken (at least one time) most frequently by the patients was opiates (67.4%), followed by dexmedetomidine (50%), benzodiazepines (39.5%), and propofol (9.3%), and 5.8% of the patients took a neuromuscular blocking agent. The median dexmedetomidine dose (μg) was significantly higher in the muscle wasting group (*p* = 0.017).

### Association Between Muscle Wasting, Prolonged Hospital Stay, and Nutritional Parameters

The mean energy delivered was 1,115 ± 440 kcal/d (15.5 ± 6.7 kcal/kg/d), with 53% ± 24% energy adequacy. The mean protein delivered was 71.2 ± 31.4 g/d (1 ± 0.5 g/kg/d), with 75.9% ± 35.2% protein adequacy. There was no difference in energy/protein intake or adequacy between the groups. The patients with muscle wasting had higher percentages of change in calf circumference (*p* = 0.025) (Table 2).

**Table 2:** Medical nutrition therapy and anthropometric measurements of critically ill patients with acute brain injury

Characteristics	All patients (*n* = 86)	Decrease of RFCSA		*p* value	Hospital LOS		*p* value
		≥ 10% (*n* = 49)	< 10% (*n* = 37)		≥ 21 days (*n* = 50)	< 21 days (*n* = 36)	
Medical nutrition therapy, mean ± SD							
Protein intake, g/kg/d	1 ± 0.5	1.1 ± 0.4	0.9 ± 0.4	0.085	1 ± 0.4	0.9 ± 0.5	0.237
Protein adequacy, %	75.9 ± 35.2	81.7 ± 34.8	68.4 ± 34.9	0.085	79.8 ± 32.6	70.3 ± 38.4	0.237
Energy intake, kcal/kg/d	15.5 ± 6.7	15.7 ± 6.6	15.4 ± 7.3	0.862	16.2 ± 7.3	14.5 ± 6.1	0.238
Energy adequacy, %	53 ± 24.5	54.4 ± 24.3	51.2 ± 25	0.548	55.1 ± 25.9	50.1 ± 22.4	0.341
Anthropometric measurements							
Baseline measurements							
Body mass index, mean ± SD	26.8 ± 3.7	27.2 ± 3.8	26.3 ± 3.5	0.309	26.9 ± 3.2	26.7 ± 4.3	0.843
Mid-arm circumference, mean ± SD or median (IQR)	30.7 ± 3.1	31.1 ± 3.4	30.1 ± 2.5	0.141	31.5 (28.5–33)	29.5 (28–31)	0.233
Calf circumference, median (IQR) or mean ± SD	36 (34–38)	37 (34–38)	34.5 (34–37.7)	0.214	36.1 ± 3.2	36.3 ± 4.3	0.804
Loss of measurements, median (IQR), %							
Δ mid-arm circumference	3.3 (− 3.1–6.1)	3.1 (− 3.2–5.9)	3.5 (0–6.9)	0.217	3.1 (− 3.2–6.1)	3.4 (0–6.9)	0.216
Δ calf circumference	5.2 (0–7.9)	5.4 (2.8–8.6)	3 (0–5.7)	0.025	5.3 (0–8.7)	5.3 (1.3–6.1)	0.811

*IQR* interquartile range, *LOS* length of stay, *RFCSA* rectus femoris cross-sectional area

### Factors Associated with Prolonged Hospital Stay from the Univariate and Multivariate Regression Analyses

Univariate and multivariate logistic regression analyses were used to identify the factors influencing prolonged hospital stay in critically ill patients with acute brain injury. In the univariate analyses, a significant association was found between prolonged hospital stay and acute muscle wasting (odds ratio [OR] 3.677; 95% CI 1.487–9.043; *p* = 0.005), mechanical ventilation status (OR 3.600; 95% CI 1.455–8.904; *p* = 0.006), and GCS score (OR 0.888; 95% CI 0.808–0.976; *p* = 0.014) at ICU admission. The multivariate analyses demonstrated that muscle wasting (OR 3.449; 95% CI 1.344–8.853; *p* = 0.010) was an independent risk factor for prolonged hospital stay (Table 3).

**Table 3:** The risk factors for prolonged hospital stay

Variables	Univariate analyses		Multivariate analyses	
	OR (95% CI)	*p* value	OR (95% CI)	*p* value
Age	1.0 (0.975–1.026)	0.986		
Sex, male	0.613 (0.257–1.460)	0.269		
Type of brain injury, TBI	0.857 (0.211–3.484)	0.829		
CCI	1.076 (0.804–1.439)	0.624		
APACHE-II	1.020 (0.962–1.082)	0.511		
GCS	0.888 (0.808–0.976)	0.014	0.958 (0.820–1.120)	0.592
SOFA	1.128 (0.937–1.358)	0.204		
Mechanical ventilation	3.600 (1.455–8.904)	0.006	2.443 (0.549–10.874)	0.592
Muscle wasting	3.677 (1.487–9.043)	0.005	3.449 (1.344–8.853)	0.010

*APACHE-II* Acute Physiology and Chronic Health Evaluation II, *CCI* Charlson Comorbidity Index, *CI* confidence interval, *GCS* Glasgow Coma Scale, *OR* odds ratio, *SOFA* Sequential Organ Failure Assessment, *TBI* traumatic brain injury

## Discussion

Our results demonstrated a significant decrease in the RFCSA within a week, and 57% of the patients had muscle wasting. Further, acute muscle wasting increases the risk of prolonged hospital stay in critically ill patients with acute brain injury.

In critically ill patients, the greatest loss of muscle mass occurs during the first week in the ICU [38]. Similar to our results, studies have reported a 12.5–23.2% decrease in the RFCSA from baseline in the first week [32, 39]. In line with other studies conducted with critically ill patients [40], in the present study, there was a high rate of muscle wasting in critically ill patients with acute brain injury. The mechanisms of muscle wasting in patients with acute brain injury have not been elucidated, and further studies are needed to determine the relationship between muscle wasting in the critical care period and sarcopenia during post-acute care.

There is increasing evidence that sedative, analgesic, and neuromuscular blocking agents affect neuromusculoskeletal function and are associated with the risk of ICU-acquired weakness, but there is not enough evidence to definitively establish the effect of these agents [41–43]. In the present study, we found that higher doses of dexmedetomidine were associated with acute muscle wasting, but other sedative and analgesic agents were not. Previous studies have mostly focused on the effect of drugs on the risk of ICU-acquired weakness, and it is known that one of the most important risk factors associated with ICU-acquired weakness is the length of stay in the ICU [44]. However, further studies are needed to determine the relationship between drugs and muscle wasting that occurs during the 7-to 10-day period, which is the acute period.

To date, few researchers have either reported nutrition intake or examined the effect of medical nutrition therapy on the maintenance of skeletal muscle during critical illness. Thus, how the adequacy of protein and energy intake affects muscle loss in intensive care populations remains unclear. Although energy and/or protein intake may seem to be associated with muscle loss [45, 46], there are also data reporting no impact on the loss of skeletal muscle [47, 48], as in the present study. Interestingly, two recent meta-analyses reported conflicting results on the relationship between protein intake and muscle wasting in critically ill patients [49, 50]. On the other hand, Gungor et al. [51] reported that higher calorie or protein intake was negatively correlated with the magnitude of reduction in cross-sectional muscle area, as evaluated by computed tomography in patients with acute dysphagic ischemic stroke. Future studies on this topic with a specific emphasis on personalized nutritional care, taking into account the patient population, disease severity, and being in a vulnerable population in terms of muscle loss, would be of importance to understand the nutritional interventions for improvement of clinical outcomes in critically ill patients.

Sarcopenia at ICU admission is associated with unfavorable outcomes, including longer mechanical ventilation, longer ICU and hospital stay, and increased risk of mortality [52, 53]. However, only a few studies have been reported on the effect of loss of muscle mass on clinical outcomes, and none of these studies were conducted in critically ill patients with acute brain injury. For instance, it was reported that the change in quadriceps muscle layer thickness, but not RFCSA, over the first week of critical illness was a predictor of 60-day mortality in mechanically ventilated critically ill patients [34]. Sabatino et al. [54] found a relationship between severity of quadriceps muscle thickness loss in the first 5 days of ICU stay and discharge status in critically ill patients with acute kidney injury. On the other hand, in other studies, no relationship was reported between acute muscle loss and clinical outcomes in critically ill patients [35, 55]. In the present study, muscle loss was assessed using ultrasound as a decrease in the RFCSA of ≥ 10% to define muscle wasting, and muscle wasting was predictive of prolonged hospital stay in critically ill patients with acute brain injury. Comparison of study results is difficult because there is no consensus on the optimal measurement protocol for muscle ultrasound, and image quality can be affected by factors related to the practitioner and the patient [56]. To more precisely characterize skeletal muscle wasting, integration of different parameters may be necessary. Surprisingly, the multivariate regression analyses in our study showed that mechanical ventilation status was not associated with prolonged hospital stay. However, this may have been due to the fact that we showed the mechanical ventilation status of our patients between the time points when we evaluated muscle loss.

The present study has some limitations. First, our arbitrary definition of prolonged hospital stay and our single-institution patient population may limit the generalizability of our findings to other settings and institutions. Second, energy requirements were determined using the Harris–Benedict equation, which is recommended for use in the absence of indirect calorimetry in intensive care patient populations. Third, the potential for selection bias should be considered, as primary patient selection was based on the investigator’s subjective assessment of which patients were likely to remain in the ICU for longer than a week. Finally, the observational nature of the present study does not allow any inference about causality to be made.

## Conclusions

In conclusion, muscle wasting occurs early and rapidly within the first 7 days of ICU stay in critically ill patients with acute brain injury. Muscle wasting appears to be related to unfavorable outcome in critically ill patients with acute brain injury. Future studies are required to further confirm these findings and determine an optimal strategy to specifically target modifiable risk factors to prevent muscle wasting.
